# Supplementary figures and images for: Siblings and nonparental adults provide alternative pathways to cultural inheritance in juvenile great tits
Source: PLoS Biol. 2025 Oct 9;23(10):e3003401. doi: 10.1371/journal.pbio.3003401 (PMC12510479; doi:10.1371/journal.pbio.3003401)

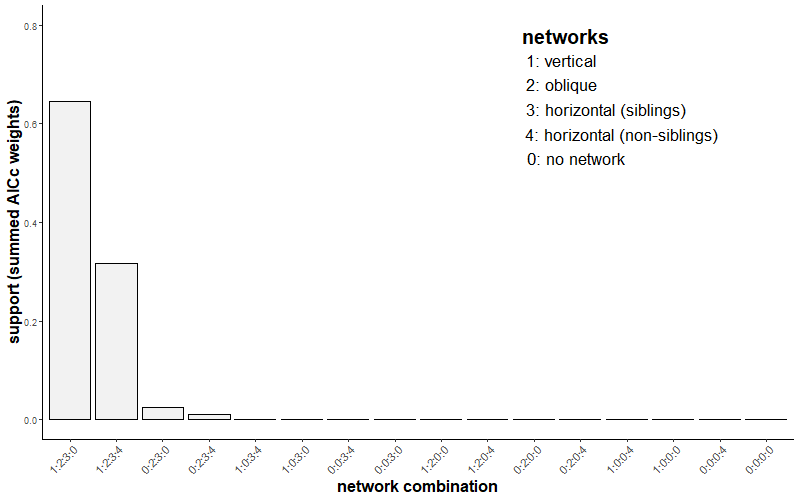

Supplement: S1 Fig — NBDA showed most support (∑wi=0.64) for models with social transmission between parents and offspring (network 1: vertical), between nonparent adults and juveniles (network 2: oblique), and among siblings (network 3: horizontal (siblings). Numerical values area available in S3 Table and at https://doi.org/10.5281/zenodo.16930533. (TIFF) [file pbio.3003401.s001.tiff]

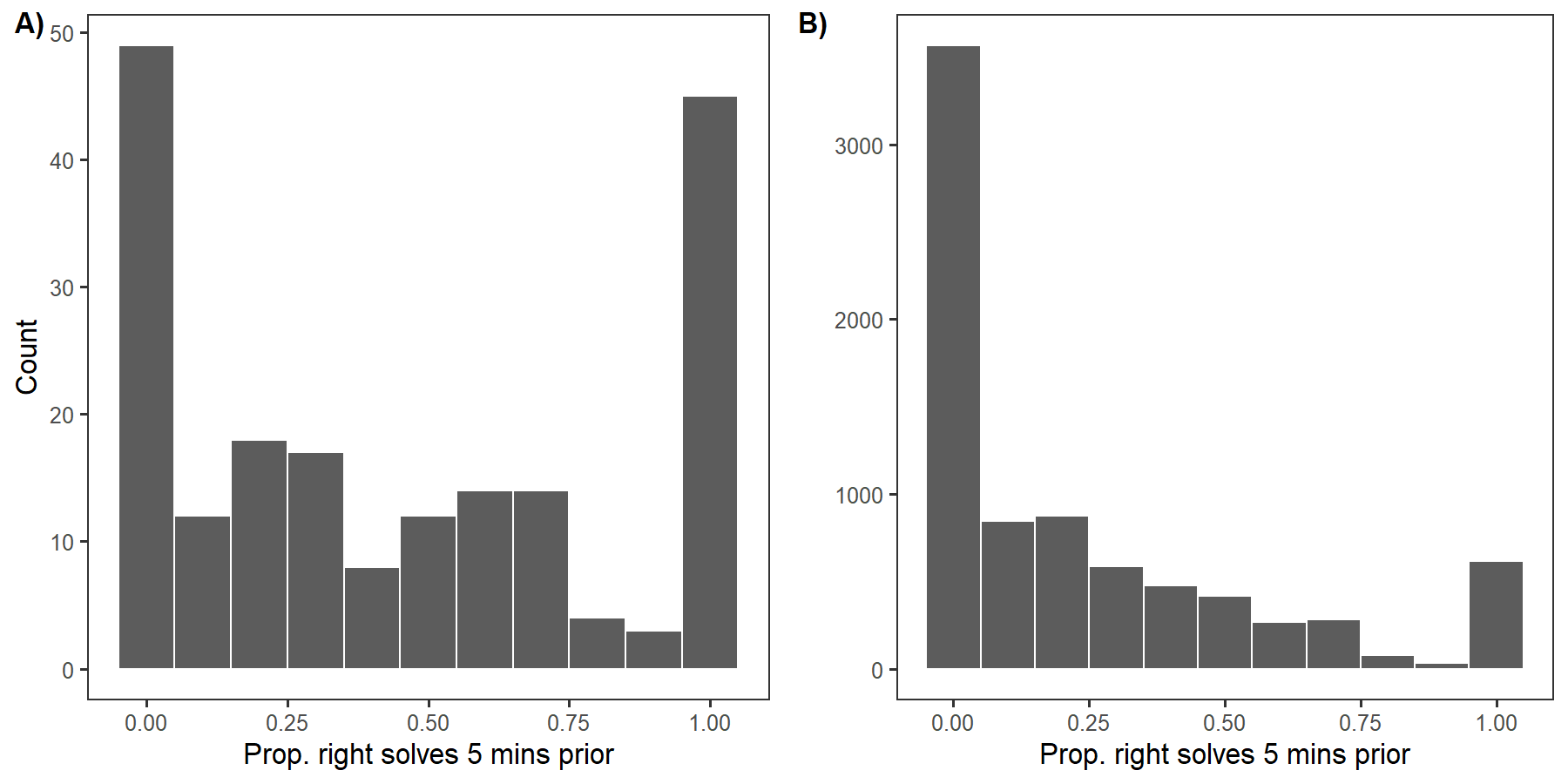

Supplement: S2 Fig — These values serve as a proxy for social information available to the juveniles at the time of solving. Data underlying this figure can be found at https://doi.org/10.5281/zenodo.16930533. (PNG) [file pbio.3003401.s002.png]
