# Supplementary material for: Siblings and nonparental adults provide alternative pathways to cultural inheritance in juvenile great tits
Source: PLoS Biol. 2025 Oct 9;23(10):e3003401. doi: 10.1371/journal.pbio.3003401 (PMC12510479; doi:10.1371/journal.pbio.3003401)
Supplement: Attachment — Submitted filename: Response to reviewers.pdf [file pbio.3003401.s012.pdf]

REPONSES TO REVIEWERS' COMMENTS:

Reviewer #1:
These authors investigated social learning strategies in juvenile great tits and found that oblique transmission—learning from non-parental individuals—is stronger than vertical transmission from parents. Interestingly, their data also show that juveniles were highly influenced by informed siblings.

The manuscript is exceptionally well-written, and the findings are of broad interest to researchers across multiple disciplines.

We thank reviewer 1 for such positive and constructive feedback. We address the raised points below:

I have only a few minor comments.
1) The first part of the Results section includes detailed methodological descriptions, but I wonder if this could be made more succinct. While I understand the Methods section is at the end of the manuscript and the authors wanted to provide context, I suggest focusing on only the essential information needed for readers to interpret the results.

We have considerably cut down these paragraphs and only retained what we believe to be essential for understanding the results. It now reads:

Both adult great tits and nestlings in two breeding populations (MI & GU) were equipped with unique passive integrated transponder (PIT) tags for remote identification using RFID. Across both populations, we monitored 306 nest boxes, from which 51 breeding pairs successfully fledged 229 chicks.

For the social learning experiment, we deployed seven automated puzzle boxes with a sliding door and diffused solutions through population for two weeks (Figure 1A). Solvers were identified via RFID antennae built into the landing perches. Of the 51 breeding pairs, 26 were both knowledgeable in solving: 13 were restricted to solve on the same side (eight right, seven left) and 13 to opposite sides. Two pairs had one knowledgeable partner each (both right), and 23 pairs had no knowledge.

After fledglings emerged from their nest boxes, puzzles were deployed for eight weeks on four days per week, recording identity and side choices of both solving adults and juveniles.

On two days per week, we additionally deployed seven RFID-equipped feeder columns (‘network feeders’) to track associations based on co-feeding events. In total, 231 tagged great tits – of which 103 were juveniles – were recorded (MI: 170 [Figure 1B]; GU: 61). Of the juveniles, 53 were knowledgeable of the puzzle box, producing 10,668 solves across the experiment.

2) Figure 3B: Is the x-axis should be "Prop of right solves 5 mins prior" instead? Also, it seems the data points do not seem to match with the fitted curve. i.e. there are many "right" choices when x = 0 and many "left" choices when x = 1, suggesting that the fitted curve should actually be reversed. I wonder if the authors accidentally switched "right" and "left" on the y-axis (as the fitted curve seems correct).

Thank you for pointing this out! The reviewer is correct on both accounts. We have swapped ‘right’ and ‘left’ in our code, and adjusted the x axis.

Reviewer #2:

This paper makes a novel and important contribution to the currently highly active field of animals' social learning and culture, frequently featured in this journal. Research on intergenerational cultural transmission has tended to focus on 'k-selected' species with long periods of juvenile dependence on parents, such as most primates and cetaceans. These also often involve a single offspring.

By contrast many other, 'r-selected' species have only a short period of dependence on parents, and there are multiple simultaneous siblings. Great tits are a good example.

This rigorous experimental study of wild great tits' learning during ontogeny clearly shows that social learning is crucial for the foraging skill required (none learned to solve the task if limited to individual exploration), but that while the first learners among siblings tended to learn from parents, or even more so from other adults, later learners tended to learn preferentially from their siblings. Figure 2 offers a nice summary. The more detailed aspect of matching the side of the foraging device observed was more dependent on the most recently observed models, rather than that preferred by the parents. All these findings are new to the field and importantly expand our understanding of the alternative pathways of cultural transmission found across the animal kingdom.

The manuscript was a delight to read in its clarity and the figures are also very clear and helpful. I judge the methods and statistical analyses to be rigorous and appropriate. The coverage of the literature in the Introduction and Discussion sections is appropriate and both are well judged.

Accordingly I have only the most minor of suggestions for a slight revision: could it be briefly explained why there were only two cases with one parent knowledgeable and one parent ignorant? (line138). So no analysis of the effects of this could sensibly be made.

In many years of reviewing I don't recall making a more positive recommendation.
END

We thank reviewer 2 for this extremely positive assessment of our study and manuscript. We are delighted to hear they have enjoyed reading and reviewing it!

Regarding the (only) two cases of only one parent being knowledgeable: The low number is a result of a combination of factors. First, when determining access restrictions for the parents, we tried to balance the different parental categories (both solving, one solving, none solving), but with a bias towards having more solving parents to have a sufficiently large number of juveniles being exposed to the puzzle box and solving behaviour. Second, there is an element of chance to who ended up in the data set, since nestling (and parental) mortality is high, and because not all birds ended up using the area enough to make the cut-off point of 10 visits to our network feeders.

We have added this information as follows:

The numbers of parents with different knowledge states reflect both our experimental design, which prioritized exposing juveniles to solving parents, and natural variation in survival and site use, which limited which breeding pairs were ultimately included in the analyses.

Reviewer #3:

Thank you for the opportunity to review this manuscript.

I judge this a well-conducted study that should be of substantial interest to the field of animal culture, and I also judge it to be appropriate for this journal. Here, the authors build on previous work concerning the spread and maintenance of puzzle box-solving behavioural traditions in great tits (Parus major).

The box-opening frequency of the parents during the dependent period did predict whether juveniles would learn to open the boxes, but other birds within the social environment (especially siblings) turned out to be of greater importance, especially once one sibling in a family group had learned from the parents. The actual method for box opening (pushing left or right) was also influenced by other birds within the social environment: siblings, and non-parental adults. Providing this level of detail about the spread of the behaviour is really fascinating, and the implications of this are discussed by the authors.

Overall, the study is well-presented, and I have relatively minor suggestions for revisions: these mostly pertain to clarity of expression/wording.

We thank reviewer 3 for the positive and thorough assessment of our manuscript. Answers to the raised points are below:

56: "...can greatly increase the efficiency with which individuals acquire novel information or behaviour, often leading to a concentration of social learning during the juvenile period [10]" - This logic is a little unclear. Would it be better to say: ...can greatly increase the efficiency with which individuals acquire novel information or behaviour. Close proxmity to parents and siblings often leads to a concentration of social learning during the juvenile period [10]?

Thank you for raising this point. They key point we were trying to make is that juveniles need to acquire many skills necessary for survival and social learning is likely an efficient way of doing so, which is why we often see a concentration of social learning during the juvenile period. We have rephrased to make this link clearer:

For efficient acquisition of behaviour, reliance on social learning is therefore often particularly pronounced during the juvenile period [1].

64: "This then in leads..." - typo? Would it be better to read "This then in turn leads to..."

Thank you for pointing out the typo. We have changed it according to the reviewer's suggestion.

66: "Yet, social learning is not restricted to species with prolonged dependence periods, especially in many bird species [17]" - the wording is a little clunky. How about "However, social learning is not restricted to species with prolonged dependence periods: this is particularly true of birds [17]" - and then perhaps a short addition clarifying that many bird species have short periods of dependence relative to their lifespan, or something, to help build the picture?

We have rephrased to clarify:

Yet, social learning is not restricted to species with prolonged dependence periods. <u>This is particularly true in many bird species who are typically characterized by short durations of parental care</u> [2].

76: Maybe expand a little? e.g. "... [20,21]. It may be better to learn from individuals they encounter in these new environments." (or something to this effect)

We have expanded as follows:

The value of parental social information is expected to rapidly decrease as young individuals gain independence and experience new environments, <u>in which they may benefit from other role models who may be more familiar with the local environment</u> [3,4].

88: Would it be more accurate to say "as multi-generational cultural traditions"?

We have changed this according to the reviewer's suggestion:

with new foraging behaviours spreading rapidly through social networks to establish as multi-generation cultural traditions [5].

94: "We currently lack information on..." might be better wording.

Changed.

100: "With onset of..." - would it be better to say "With the onsent of..." or "On the onset of..."

Changed to:

With the onset of independent feeding....

101: "Previous work used cross-fostering experiments..." - possibly reword to "Previous cross-fostering experiments, where eggs were swapped between great tits and blue tits (Cyanistes caeruleus), found that..."

Changed according to the reviewer's suggestion.

105 onwards: Do you mean 'vertical learning' solely to refer to parent -> offspring social learning, and no other form of learning? It might be worth clarifying "...and subsequently copied behaviour from other individuals in their foster species after reaching independence, but not necessarily from their foster parents."

Yes, we refer to vertical learning as learning exclusively from parents. We have clarified this as follows:

Alternatively, however, developing individuals could have imprinted on the 'wrong' species during a sensitive period and subsequently copied behaviour from other individuals in their foster species <u>other than their foster parents</u> after reaching independence.

135 "Solver's identifies were identified" - maybe "Solver's identities were determined" would be better to avoid redundancy.

In response to reviewer 1, we have rephrased large parts of this paragraph. It now reads:

Solvers were identified via RFID...

140 Would "the behaviour continued to diffuse" or "we allowed the behaviour to continue to diffuse" be more accurate?

In response to reviewer 1, this particular sentence has been removed as we made this part more concise.

163 Local enhancement, to my understanding, is still a form of social learning (and so would not preclude the behaviour from being culturally transmitted?). What would the implication of a local enhancement effect be for these results? Perhaps this just isn't clear to me why local enhancement should be controlled for in this section - unless you are specifically wanting to determine that the learning must be observational. I would suggest making this clearer/justifying it.

Yes we agree, that is the point we were trying to make, that comparing scrounging and knowledgeable parents allows us to say something more about the learning mechanisms. We have clarified as follows:

Inclusion of the number of scrounges allowed us to distinguish between observational learning – i.e. offspring learn to solve because they are exposed to the motor-patterns – and a local enhancement effect, if simply being attracted to the puzzle box site by scrounging parents is sufficient for juveniles to learn to solve.

And in the discussion:

Meanwhile, parental scrounging behaviour during dependence did not predict the offspring probability of learning to solve, indicating that local enhancement to the puzzle site alone is not sufficient to acquire knowledge <u>*but instead suggestive of an effect of observational learning*</u>*.*

164 onwards:
I know this is said in the methods (birds were considered knowledgeable after three solves) but this could use defining here as well as in the methods

We added this to the introductory paragraph of the result where we briefly discuss the experimental setup.

Juveniles were considered knowledgeable if they produced at least 10 solves.

195: most of the first siblings to learn, learned from non parent adults! subsequent learners mostly learned from siblings.

Indeed, we have highlighted this in the discussion (see below).

211: Would this be better as "In a final set of models" or "In the last set of models"?

Changed to:

In a final set of models,

245: Should this be "manipulate the knowledge state"?

Indeed, we have added ‘the’.

255: Should this be "difference in" or "distinction between"?

We have rephrased to ‘difference’.

256 onwards: oblique learning from non-parental models seemed to be higher than from parental models (line 185 onwards) - but this isn't really discussed at all. I think this should be highlighted and considered in the discussion, too.

We have highlighted this in the discussion more prominently:

While the first learners in each sibling cohort learned primarily from non-parental adults, subsequent learners within the sibling cohort almost exclusively learned from their already knowledgeable siblings. Meanwhile, only few seemed to have learned directly from parents.

267: Is this a feature common to great tits? In general, I think there could be some more description of great tit background behaviour/life history, possibly in the introduction. i.e., you mention this behaviour from line 284 onwards, but it might be better in the introduction (to build the story behind this work).

The continued preferred association with siblings even after reaching independence from parents has been demonstrated in one of our recent studies. We have added the reference in the discussion, and extended the introduction as follows:

With the onset of independent feeding, they reach full independence from parents and integrate into local flocks, although they may continue to prefer to associate with siblings and peers over adults [6].

Reviewer #4:

This study presents experimental evidence for the drivers of information use and foraging decisions in newly-independent songbirds. This study is initially similar in outlook to a previous work (i.e. studying information use and foraging decisions during early life in a short-lived passerine with limited parental care - see Franks et al. 2020); however, the present study offers an elegant and more extensive study design to develop this area further, and I would like to commend the authors for this. I also found this paper very clear to read. As a result, I have only a few minor comments to raise.

We thank reviewer 4 for this positive assessment of our study and the value it adds to the study conducted by Franks et al 2020.

L143: How were the different solutions mixed across the population to ensure juveniles would encounter a variety of solutions once fledged - i.e. how do juveniles disperse across the study site? Perhaps just worth a mention here or in Methods.

We extracted the histograms of the proportion of right solves juveniles experienced in the 5 minutes immediately prior to solving for a) their first solving day and b) across all experimental days. While the available social information is biased towards occurring on one side, there is overall a good mix of information they receive. The shift towards more solves occurring on the

left across all experimental days is likely due to the initial conformist tendencies of the juveniles as they started solving (slight initial preference for left because majority of birds solved left) and subsequent reliance on personal information (staying left), which in turn provided social information to other naive juveniles, overall leading to a left bias across the population despite certain adults still being restricted to solve right.

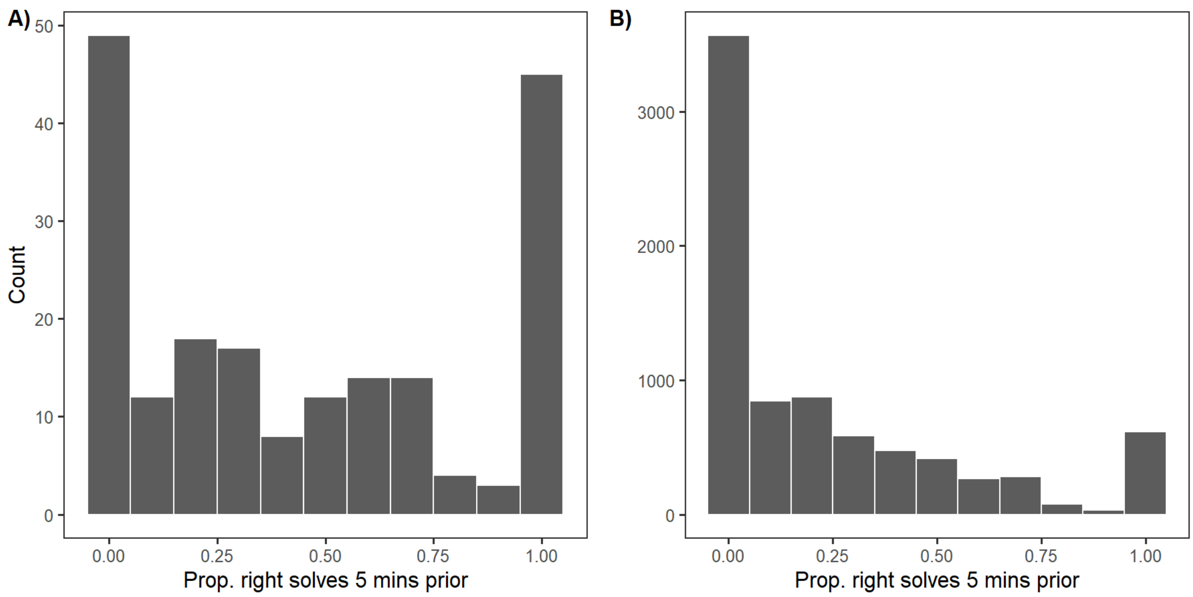

We have added the histogram as an additional figure to the supplementary material and added a section to the manuscript to refer to the figure:

As a proxy for social information available to juveniles, for each solve, we extracted the proportion of solves occurring on the right side in the five minutes immediately prior to solving. While there was evidence for local biases of solves occurring either on the left or right, juveniles were exposed to a wide range of social information on side choices (Figure S2).

L265-266: I'm just wondering about the mechanism here - is this likely to be a learned aspect, e.g. experienced more feeder use with parents, or more innate e.g. inheritance of traits which mean they are more likely to interact with feeders (and thus potentially solve)? Could a small discussion of this be added?

The fact that most juveniles only learned to solve the puzzle if they were hatched to solving parents suggests that there is likely some observational learning occurring, but that they do not fully master the behaviours until they have reached independence from their parents. Of course, this could also potentially indicate genetic inheritance of traits required for solving (e.g. of underlying cognition). However, we consider this unlikely because previous work on this species has shown that the major predictor of learning within a single generation is social network position (i.e. opportunity for social learning), and that >80% of the population can acquire this behaviour through social learning.

We have expanded as follows:

Our study suggests that early exposure to the foraging behaviour of parents during dependence may facilitate learning, likely through observational learning of general motor patterns. Yet, full acquisition of foraging techniques may largely occur after juveniles have reached independence

from their parents but are still associating with siblings [6].

L275 and L303: correct "hihis" to "hihi" - the plural works in the same way as "sheep"!

Thank you for pointing this out! We changed it accordingly.

L302: I think the vervet monkey study [50] showed that migrant males continued to prefer the dominant food colour later on too - i.e. it persisted after their initial choice, even with some personal experience (in that paper's Supplementary Information). There, conformity was (a) "a disposition to copy the choices of a majority of others in a social group", and (b) "a willingness to subjugate one's own countervailing knowledge in matching the majority's choice". So perhaps this doesn't quite reflect what you found, if great tits were less likely to conform later on?

Thank you for pointing out this subtlety. In light of this, we have changed this reference to an example from the primate literature where chimpanzees tend to copy more when uncertain.

Kendal R, Hopper LM, Whiten A, Brosnan SF, Lambeth SP, Schapiro SJ, Hoppitt W. 2015 Chimpanzees copy dominant and knowledgeable individuals: implications for cultural diversity. *Evol. Hum. Behav.* **36**, 65–72. (doi:10.1016/j.evolhumbehav.2014.09.002)
